# Trends and hotspots in gut microbiota and neonatal necrotizing enterocolitis: a bibliometric analysis

**DOI:** 10.3389/fcimb.2026.1684491

**Published:** 2026-05-21

**Authors:** Jing Feng, Xu He, Rui Li, Cheng Chen, Zhangbin Yu, Li Zhang

**Affiliations:** 1 Department of Neonatology, Longgang District Maternity and Child Healthcare Hospital of Shenzhen City (Longgang Maternity and Child Institute of Shantou University Medical College), Shenzhen, China; 2 Department of Neonatology, Shenzhen People’s Hospital, The Second Clinical Medical College, Jinan University, The First Affiliated Hospital, Southern University of Science and Technology, Shenzhen, China

**Keywords:** bibliometric analysis, CiteSpace, gut microbiota, neonatal necrotizing enterocolitis, VOSviewer

## Abstract

**Background:**

Neonatal necrotizing enterocolitis (NEC) is one of the most life-threatening gastrointestinal emergencies in preterm infants. Accumulating evidence has identified gut microbiota dysbiosis as a key driver of NEC pathogenesis. Probiotics/prebiotics and bioactive components in breast milk can reduce NEC risk by modulating microbiota balance. With microbial intervention strategies becoming a research hotspot, publications in this field have surged over the past 5 years. However, no studies have yet systematically mapped its knowledge evolution and collaboration networks.

**Objective:**

This study pioneers the use of bibliometric methods to systematically analyze global research trends, core contributors (countries, institutions, authors), and emerging frontiers in gut microbiota and NEC research from 2005 to 2024, aiming to provide strategic guidance for future translational studies.

**Methods:**

A total of 1,011 English-language articles were screened from the Web of Science Core Collection. Software tools, including VOSviewer, CiteSpace, and Pajek, were employed to analyze publication trends, country/region and institution collaboration networks, journal co-citations, keyword clustering, and keyword bursts.

**Results:**

Annual publications increased from 5 in 2005 to a peak of 120 in 2023, with cumulative citations reaching 52,330 and an h-index of 115. The United States led in output (424 publications, 41.9%) and total citations (30,446). China ranked second in publications (158 articles) but exhibited lower average citations per article (22.65). The University of California System (65 publications) and the State University System of Florida (51 publications) were the most productive institutions. Mark A. Underwood (31 publications) focused on multi-omics mechanisms, while Josef Neu achieved the highest average citations per article (107.88) for his work on microbe–host interactions. “Nutrients” published the most articles (49), while “Microbiome” (impact factor 12.7) demonstrated the highest influence. Research hotspots evolved from early-stage microbiota composition analysis to mid-phase mechanism exploration and probiotic intervention evidence synthesis, shifting recently toward clinical translation, intestinal barrier repair, and stem cell therapy.

**Conclusion:**

Research on gut microbiota and NEC shows sustained growth, with trends shifting from microbiota structure description to multi-omics mechanistic exploration and accelerating clinical translation.

## 1 Introduction

Neonatal necrotizing enterocolitis (NEC) is the most prevalent life-threatening gastrointestinal emergency in preterm infants, particularly affecting very low birth weight (VLBW) infants. It occurs in 5%–10% of preterm infants and is associated with mortality rates as high as 20%–30%. Survivors often face long-term sequelae such as intestinal strictures and neurodevelopmental impairments (Beharry et al., 2023; Karaca and Takcı, 2025). Despite advancements in perinatal medicine, the pathogenesis of NEC remains incompletely elucidated, and its global incidence has not significantly declined over the past decade, highlighting the urgent need for effective prevention and treatment strategies (Kaplina et al., 2023a; Snyder and Hunter, 2023).

Recent studies have confirmed that gut microbiota dysbiosis serves as a core driver in the pathogenesis and progression of NEC. In preterm infants, delayed microbial colonization, reduced diversity, and pathogenic overgrowth (*Klebsiella*, Enterobacteriaceae) compromise intestinal mucosal barrier function, trigger aberrant immune-inflammatory responses, and ultimately lead to enterocyte necrosis (Aziz et al., 2022; Karaca and Takcı, 2025). External factors such as hypoxia and antibiotic exposure further exacerbate microbial imbalance, establishing a vicious cycle of “microbiota–metabolite–gut barrier” disruption (Kaplina et al., 2023a; Xia and Claud, 2023). Probiotics/prebiotics—particularly *Bifidobacterium* and *Lactobacillus* complexes—along with bioactive breast milk components such as human milk oligosaccharides (HMOs) and lactoferrin mitigate dysbiosis and prevent NEC by regulating microbiome development (Davis et al., 2020; Masi et al., 2024).

As microbial intervention strategies have become a research hotspot in NEC studies, publications in this field have surged over the past 5 years. However, no studies have yet systematically mapped the knowledge structure evolution and collaboration networks within this domain (Beharry et al., 2023). Despite the growing body of evidence linking gut microbiota dysbiosis to NEC pathogenesis, significant knowledge gaps remain. First, the causal relationship between specific microbial taxa and NEC is still debated, as most studies are associative rather than mechanistic (Pammi et al., 2017). Second, although probiotic trials have shown promise, optimal strains, dosages, and treatment durations have not been established, and some randomized controlled trials have reported neutral or even adverse outcomes (Chang et al., 2017). Third, the translational gap from animal models to clinical practice persists, particularly regarding the safety and efficacy of interventions such as fecal microbiota transplantation in preterm infants (Brunse et al., 2022). Highlighting these gaps is essential for guiding future research priorities. This study employs bibliometric methods to quantitatively analyze global trends, core contributing institutions, and research frontiers in gut microbiota and NEC research from 2005 to 2024, aiming to map the knowledge landscape and provide strategic guidance for future translational research.

## 2 Materials and methods

### 2.1 Data sources

We systematically searched the Web of Science Core Collection (WoSCC) database for bibliometric analysis, covering the period from 1 January 2005 to 31 December 2024. To ensure reliability, the data were independently screened by two authors according to predefined inclusion and exclusion criteria. The search strategy was as follows: TS = (“gut microbiota” OR “intestinal microbiota” OR “fecal microbiota” OR “gastrointestinal microbiota” OR “gut microbiome” OR “intestinal microbiome” OR “fecal microbiome” OR “gastrointestinal microbiome” OR “intestinal bacteria” OR “gut bacteria” OR “fecal bacteria” OR “gastrointestinal bacteria” OR “intestinal flora” OR “gut flora” OR “fecal flora” OR “gastrointestinal flora” OR “gut microflora” OR “intestinal microflora” OR “fecal microflora” OR “gastrointestinal microflora”) AND (“necrotizing enterocolitis” OR “enterocolitis, necrotizing”).

### 2.2 Data collection

**Figure 1:**
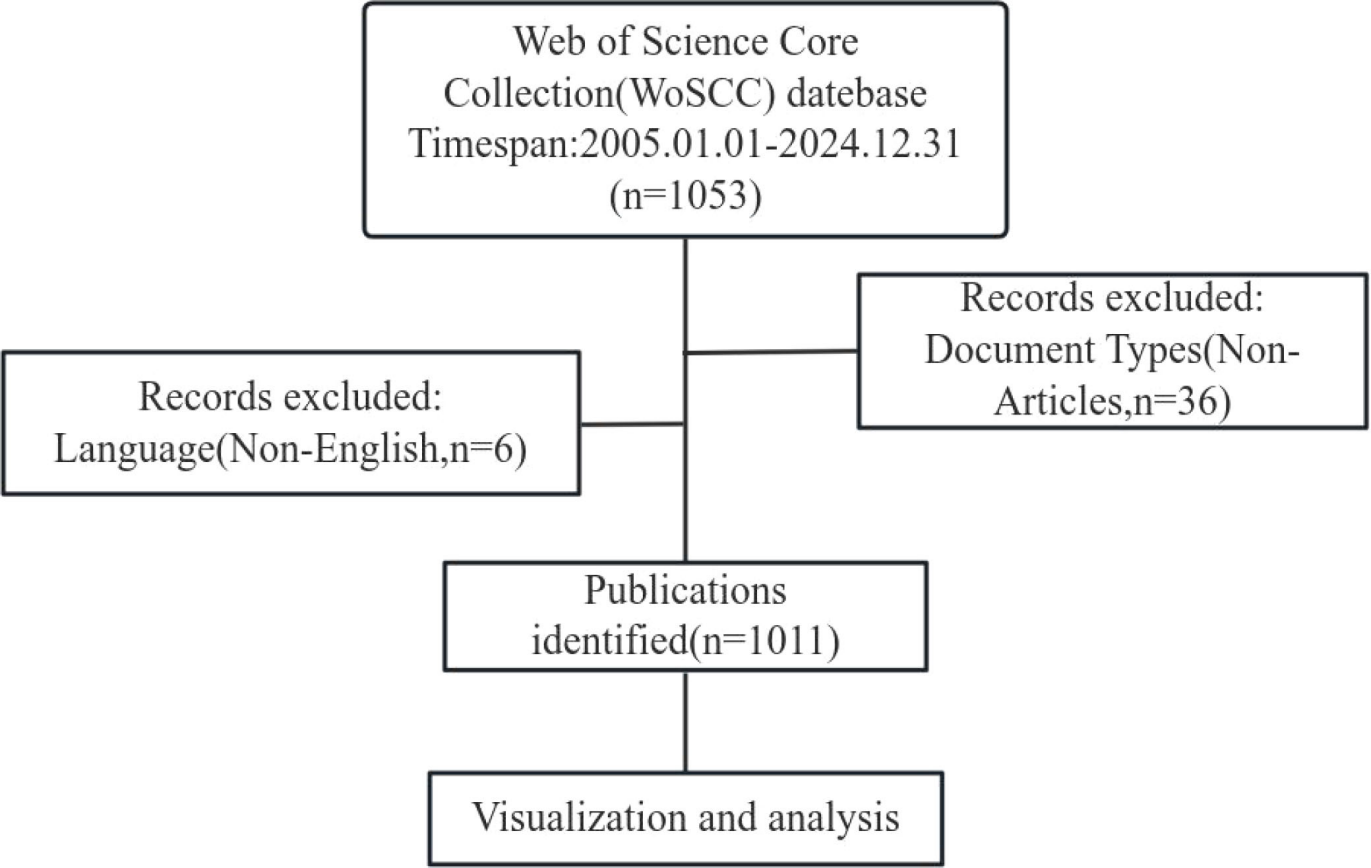
Flowchart of the literature screening.

A total of 1,053 articles were retrieved from the Web of Science database. After excluding 36 non-articles and 6 non-English articles, with no duplicates identified during further deduplication, 1,011 published articles were ultimately included (631 research articles and 380 review articles). The overall workflow of the analysis is shown below (**Figure 1**).

Raw data extracted from the WoSCC database encompassed article titles and citations, publication year, countries/regions, affiliations, authors, journals, references, keywords, 2024 journal impact factor (IF), 2024 journal citation reports (JCR) categories, and h-index.

### 2.3 Data cleaning

Prior to bibliometric analysis, we performed meticulous processing of raw data. Geopolitical entities sharing national sovereignty were consolidated; for example, Scotland, England, Wales, and Northern Ireland were combined under the “United Kingdom.” Country abbreviations were standardized; for example, “Peoples R China” was replaced with “China” and “USA” was replaced with “United States.” Keywords underwent normalization through the substitution of synonyms with controlled vocabulary. These procedures ensured data accuracy, which is critical for reliable bibliometric analysis.

### 2.4 Bibliometric analysis

Bibliometric analysis is a quantitative approach that examines large-scale publication data to uncover patterns in scientific output, collaboration networks, and emerging research fronts. Unlike systematic reviews that synthesize clinical outcomes, bibliometric studies do not evaluate intervention efficacy directly. Instead, they identify thematic clusters, citation bursts, and collaborative structures, which can inform researchers about the field’s intellectual structure and potential blind spots. In this study, we used VOSviewer for co-occurrence and co-citation mapping, CiteSpace for temporal evolution and burst detection, and Pajek for network community detection. CiteSpace, developed by Chen (2004), is a software tool designed for visualizing and analyzing citation networks within scholarly literature. VOSviewer, created by Nees Jan van Eck et al (van Eck and Waltman, 2010), facilitates rapid acquisition of macroscopic perspectives on large-scale bibliographic data, enabling identification of research hotspots, emerging trends, and key literature and research directions relevant to specific scientific inquiries. Pajek was used to perform large-scale network structure analysis and community detection in collaboration networks, complementing the visualization of VOSviewer and CiteSpace.

## 3 Results

### 3.1 Annual publication trends

**Figure 2:**
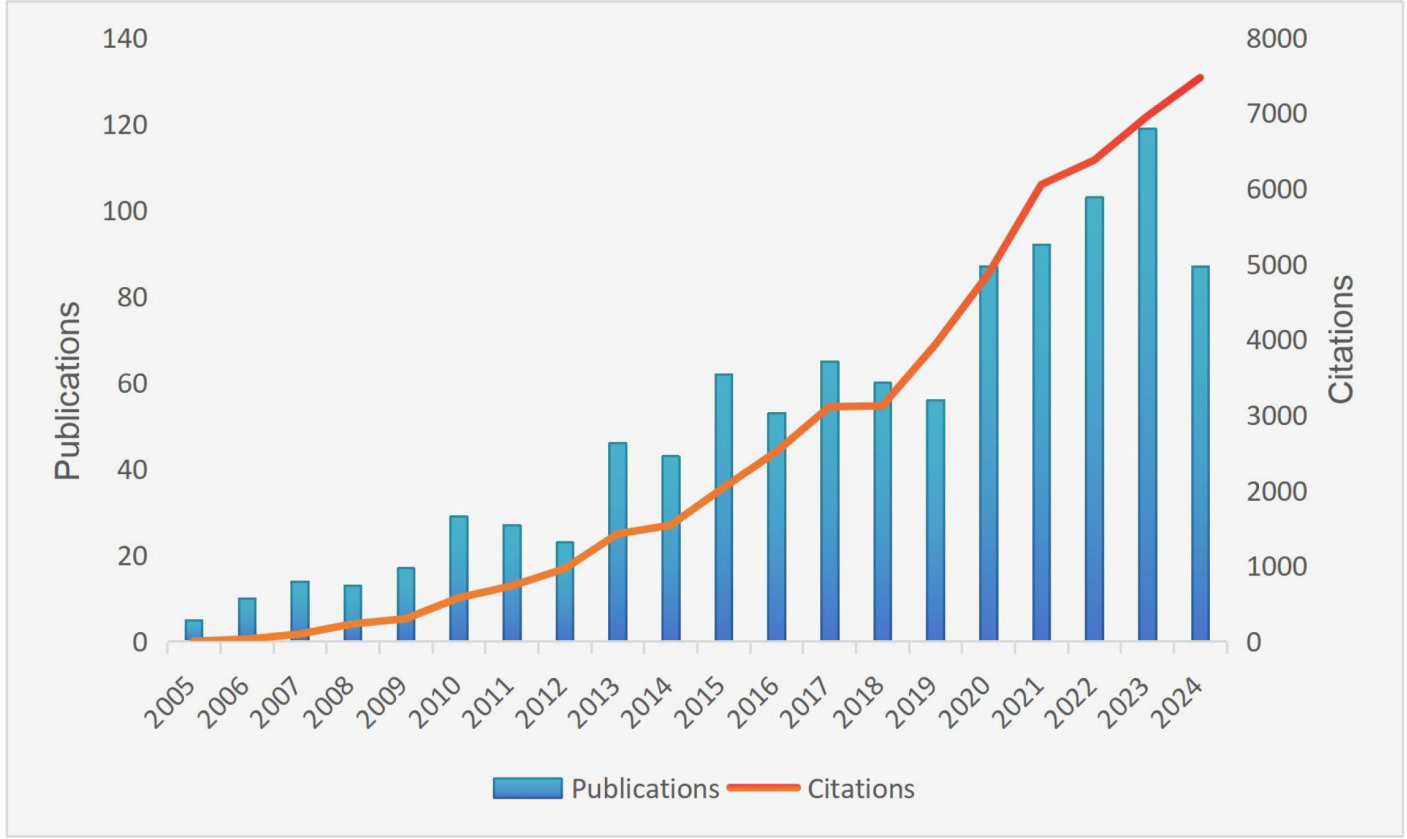
Annual publication volume and citation frequency of literature in gut microbiota and NEC research from 2005 to 2024.

Publication volume and citation frequency serve as critical indicators of research field development and growth potential. As shown in **Figure 2**, among the 1,011 included articles, annual publications demonstrated a marked upward trajectory—rising gradually from 5 in 2005 to a peak of 120 in 2023. Citation analysis revealed cumulative citations of 52,330 for all included articles, with an accelerating annual increase culminating in 7,469 citations within 2024 alone. These metrics reflect intensifying scholarly interest in gut microbiota and NEC research in recent years. The collective h-index of 115 further confirms the field’s significant impact.

### 3.2 Country/region analysis

**Figure 3:**
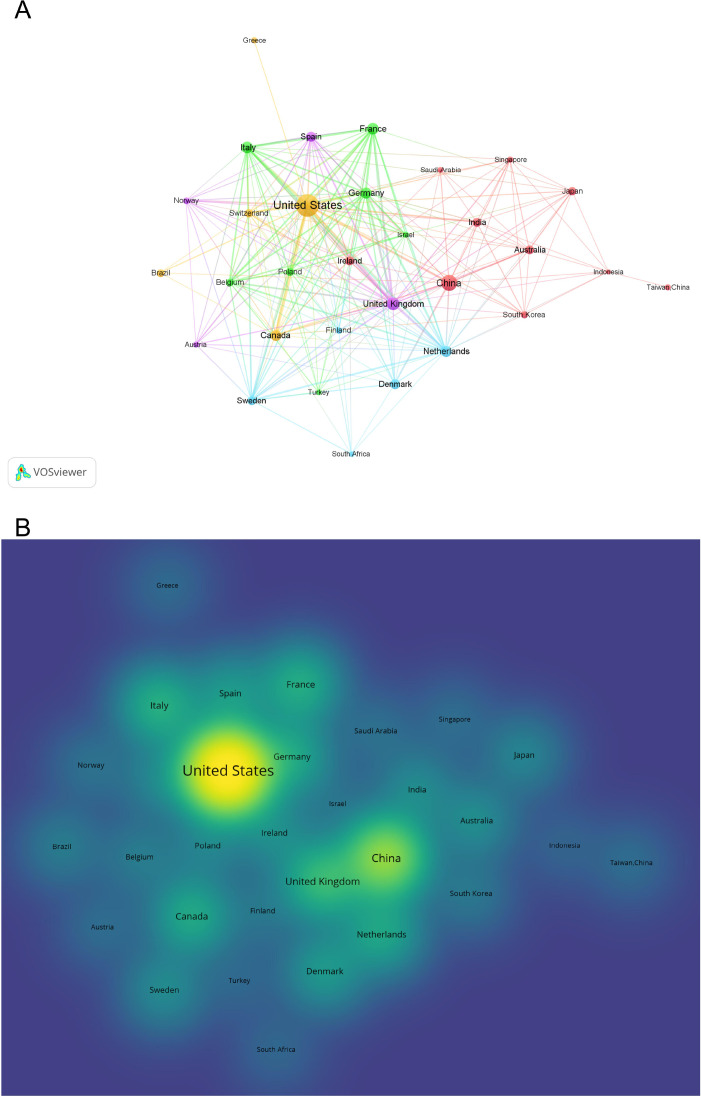
**(A)** Collaboration map of countries/regions. **(B)** Density visualization in gut microbiota and NEC research.

Across the 1,011 included publications, 65 countries/regions contributed to gut microbiota and NEC research. **Table 1** lists the top 10 productive countries/regions. The United States dominated with 424 publications (41.9%), significantly exceeding other nations in total citations (30,446) and collaborative network strength (total link strength: 165), confirming its role as the core knowledge innovation hub. Although China ranked second in output (158 publications), its citations per article (22.65) and total citations (3,579) lagged substantially behind Western counterparts, highlighting the need to enhance research quality and global impact. The collaboration map in **Figure 3** reveals regional partnerships, with European nations like the UK and Italy forming a distinct secondary cluster through intensive cooperation.

**Table 1:** The top 10 countries by number of publications.

Rank	Country	Documents	Citations	Total link strength
1	United States	424	30,446	165
2	China	158	3,579	42
3	United Kingdom	77	4,707	119
4	Italy	60	6,468	91
5	France	56	3,698	86
6	Canada	52	4,129	48
7	Netherlands	49	3,140	87
8	Germany	47	3,784	95
9	Denmark	36	1,356	29
10	Spain	36	3,742	47

### 3.3 Institutional analysis

**Figure 4:**
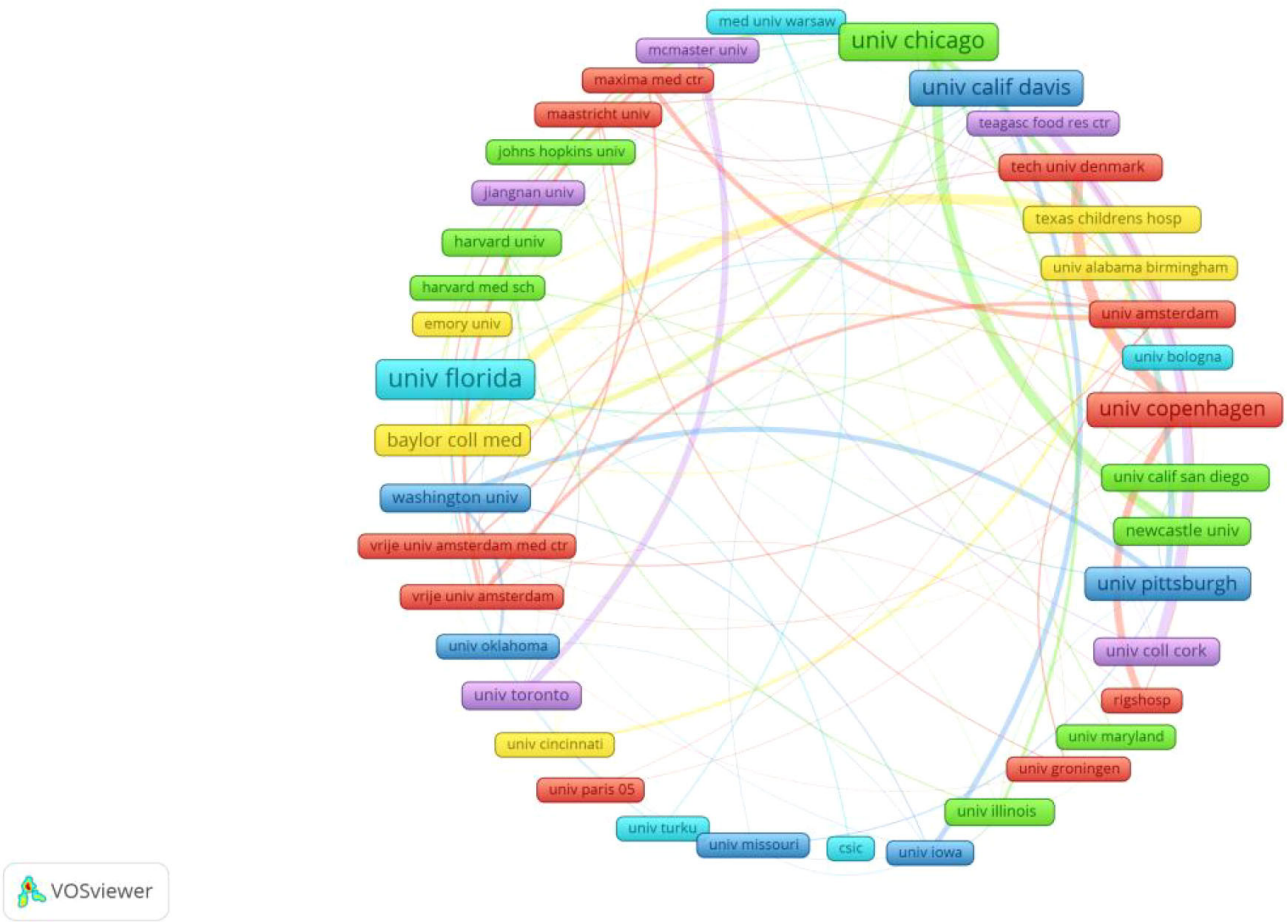
Institutional cooperation map in gut microbiota and NEC research.

Leading research institutions provide the foundation for high-quality publications. **Table 2** ranks the top 10 institutions by publication volume. The University of California System leads with 65 publications, also ranking first in total citations (5,247), average citations per article (80.72), and h-index (36). Notably, 8 of these top 10 institutions are American, with one Danish and one French institution, underscoring the United States’ dominance in this field. Institutional collaboration analysis using VOSviewer reveals research partnerships through network mapping, where thicker connecting lines denote stronger collaborative ties (**Figure 4**). A particularly robust partnership exists between the University of California, Davis and the University of Chicago.

**Table 2:** The top 10 affiliations by number of publications.

Rank	Affiliations	Count	Total citations	Average citations	h-index
1	University of California System	65	5,247	80.72	36
2	State University System of Florida	51	3,861	75.71	29
3	University of Florida	44	3,163	71.89	27
4	University of Chicago	40	2,503	62.58	24
5	University of California Davis	38	2,234	58.79	25
6	Harvard University	35	3,663	104.66	26
7	Baylor College of Medicine	33	3,051	92.45	25
8	University of Copenhagen	32	1,269	39.66	19
9	Pennsylvania Commonwealth System of Higher Education PCSHE	31	1,573	50.74	19
10	Universite Paris Cite	30	1,753	58.43	19

### 3.4 Analysis of journal publications

**Figure 5:**
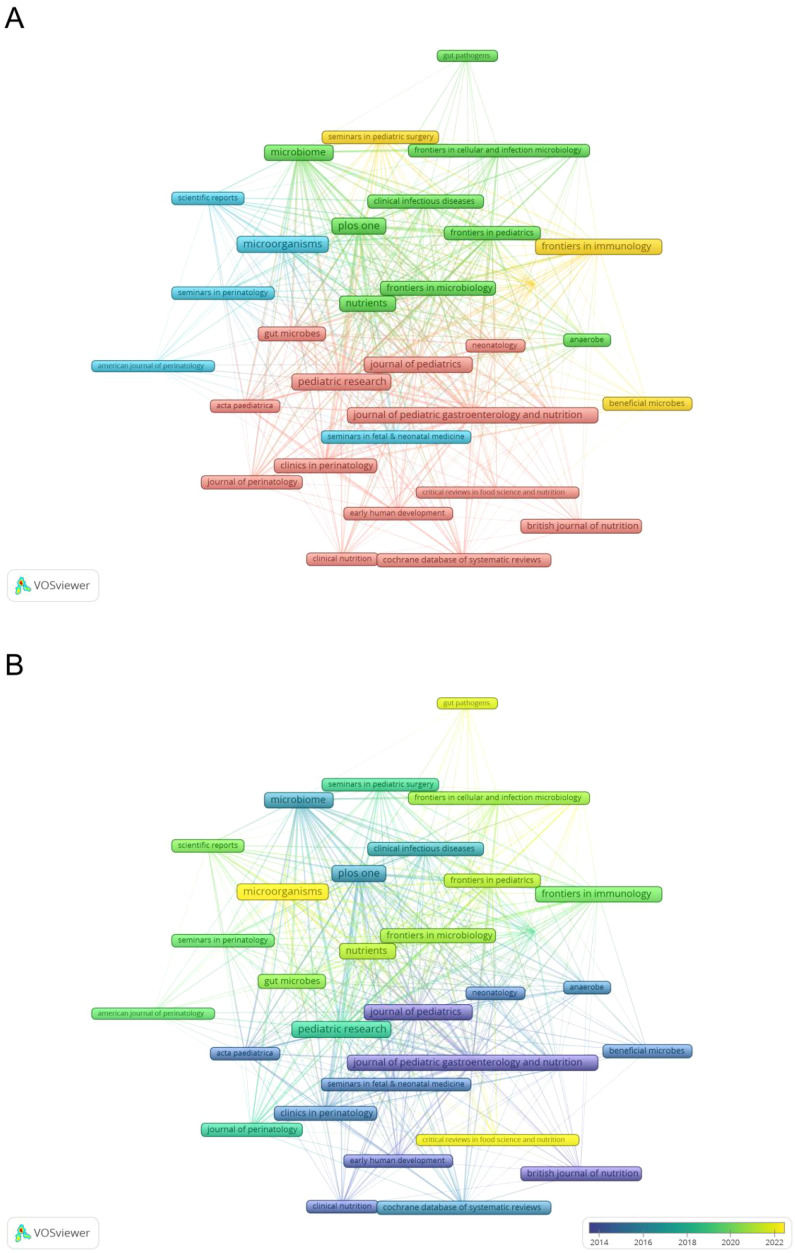
**(A)** Network map of co-cited journals. **(B)** Temporal evolution of co-cited journals in gut microbiota and NEC research.

Visual analysis of journal publications identified 359 journals that have published articles on NEC and gut microbiota-related research. Among these, 33 journals published more than six articles each. **Table 3** lists the top 10 journals ranked by publication volume, with Nutrients (49 articles) being the most prolific, followed by Pediatric Research (34 articles) and Frontiers in Microbiology (32 articles). *Microorganisms* exhibited the highest total citations (2,486) and the highest average citations per article (130.84). Six of these journals are ranked as JCR Q1, among which *Microbiome* holds the highest impact factor (12.7). As shown in **Figure 5**, journals in the same color represent clusters with closely related research themes. The connecting lines reflect the strength of journal co-citation relationships, with the extensive network revealing the interdisciplinary nature of research collaborations. **Figure 5**, the timeline map, highlights prominent journals at different stages, indirectly indicating shifts in the field’s research hotspots.

**Table 3:** The top 10 journals by number of publications.

Rank	Journal	Publications	Total citations	Average citations	h-index	IF	JCR
1	Nutrients	49	1,962	40.4	25	5	Q1
2	Pediatric Research	34	2,170	63.82	21	3.1	Q1
3	Frontiers in Microbiology	32	1,065	33.28	19	4.5	Q1
4	Journal of Pediatric Gastroenterology and Nutrition	30	1,665	55.5	21	2.6	Q2
5	PLOS One	30	2,101	70.03	22	2.6	Q2
6	Frontiers in Pediatrics	25	654	26.16	12	2	Q2
7	Gut Microbes	24	1,152	48	19	11	Q1
8	Frontiers in Immunology	23	1,887	82.04	17	5.9	Q1
9	Microorganisms	19	2,486	130.84	8	4.2	Q2
10	Microbiome	12	1,506	125.5	12	12.7	Q1

### 3.5 Author analysis

**Figure 6:**
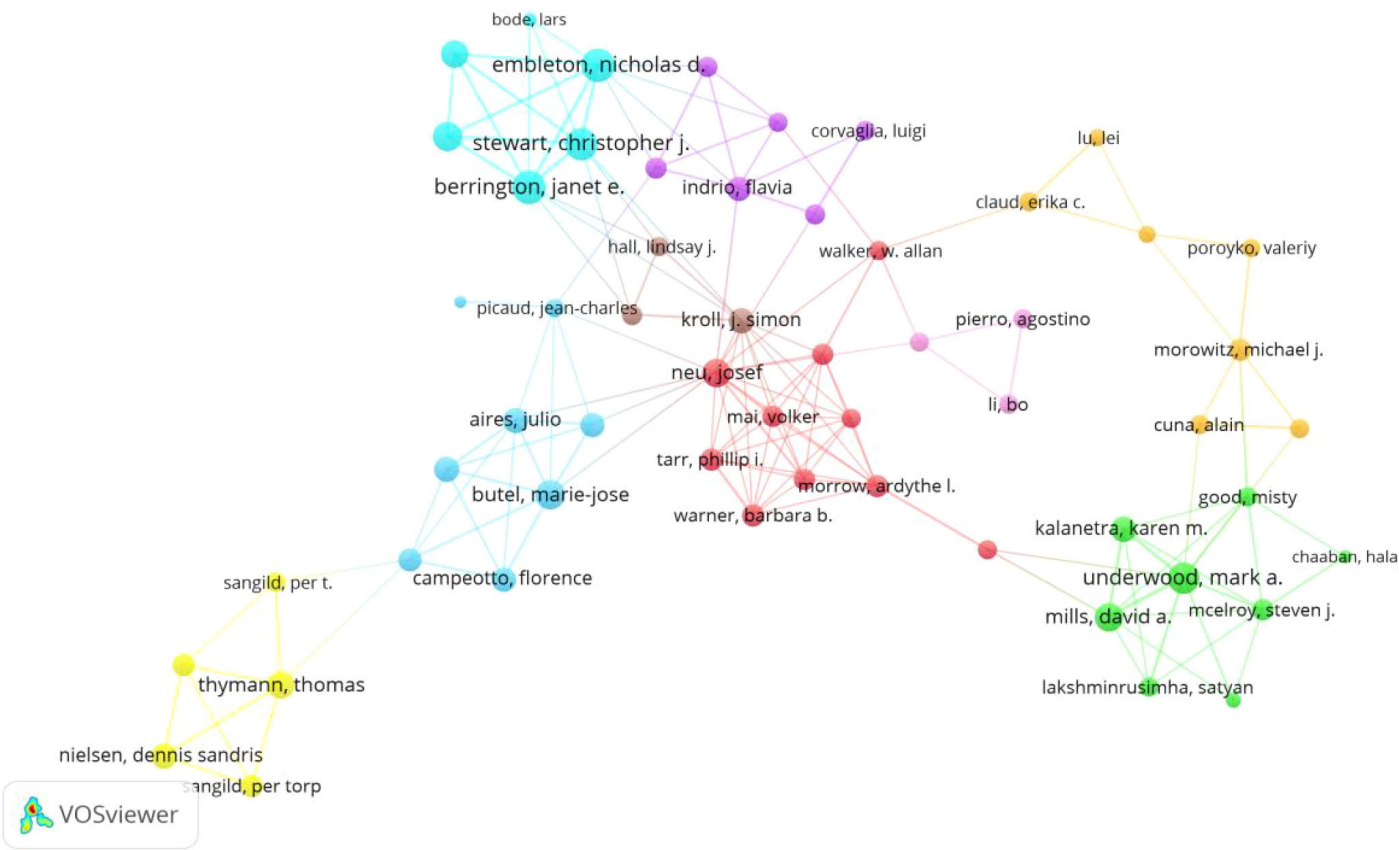
Co-authorship network in gut microbiota and NEC research.

**Table 4** displays the top 10 most published authors in this field, along with their publication counts, total citations, average citations per article, h-index, and affiliated institutions. Professor Mark A. Underwood from California State University Sacramento ranks first, with 31 publications, 2,066 total citations, and an h-index of 23. His research not only validated the significant efficacy of specific probiotics (*Bifidobacterium longum* subsp. *infantis*) in reducing gut inflammation and NEC risk through the metabolism of HMOs (Larke et al., 2022; Mills et al., 2023) but also confirmed that bioactive components in breast milk (such as HMOs and lactoferrin) protect the intestinal barrier by promoting beneficial bacterial colonization, inhibiting pathogens, and modulating the Toll-like receptor 4 (TLR4) signaling pathway (Underwood et al., 2020; Zeinali et al., 2022). Professor Josef Neu from the University of Florida ranks second with 26 publications. Despite ranking second in output, his work demonstrates exceptional impact, with an average citation rate of 107.88 per article, establishing him as a high-impact researcher. His studies focus on the role of microbe–host interactions in NEC pathogenesis and advocate for precision nutrition interventions to remodel gut microbiota balance for improved preterm infant outcomes (Rozé et al., 2017; Neu and Pammi, 2018). Professor W. Allan Walker from Harvard Medical School has published 15 articles, with the highest average citation count among the top 10 authors (135.47). His research focuses on neonatal gut microbiota establishment, immune development, and microecological intervention strategies (Houghteling and Walker, 2015; Meng et al., 2020). He has also participated in formulating international consensus guidelines for probiotic applications (Floch et al., 2011, Floch et al., 2015). Additionally, collaborative networks among these authors are extensive (**Figure 6**).

**Table 4:** The top 10 authors by number of publications.

Rank	Author	Documents	Citations	Average citation	h-index	Affiliations
1	Underwood, Mark A.	31	2,066	66.65	23	California State University Sacramento
2	Neu, Josef	26	2,805	107.88	21	University of Florida
3	Sangild, Per Torp	23	988	42.96	16	Copenhagen University Hospital
4	Morowitz, Michael	22	1,670	75.91	16	University of Pittsburgh
5	Janet E. Berrington	20	1,214	60.7	16	Newcastle University
6	Stewart, Christopher	20	1,179	58.95	16	Newcastle University
7	Claud, Erika	20	1,288	64.4	14	University of Chicago
8	Dr. Nicholas Embleton	19	1,168	61.47	15	Newcastle University
9	Butel, M.	16	944	59	12	Universite Paris Cite
10	Walker, WA	15	2,032	135.47	14	Harvard Medical School

### 3.6 Keyword analysis

**Figure 7:**
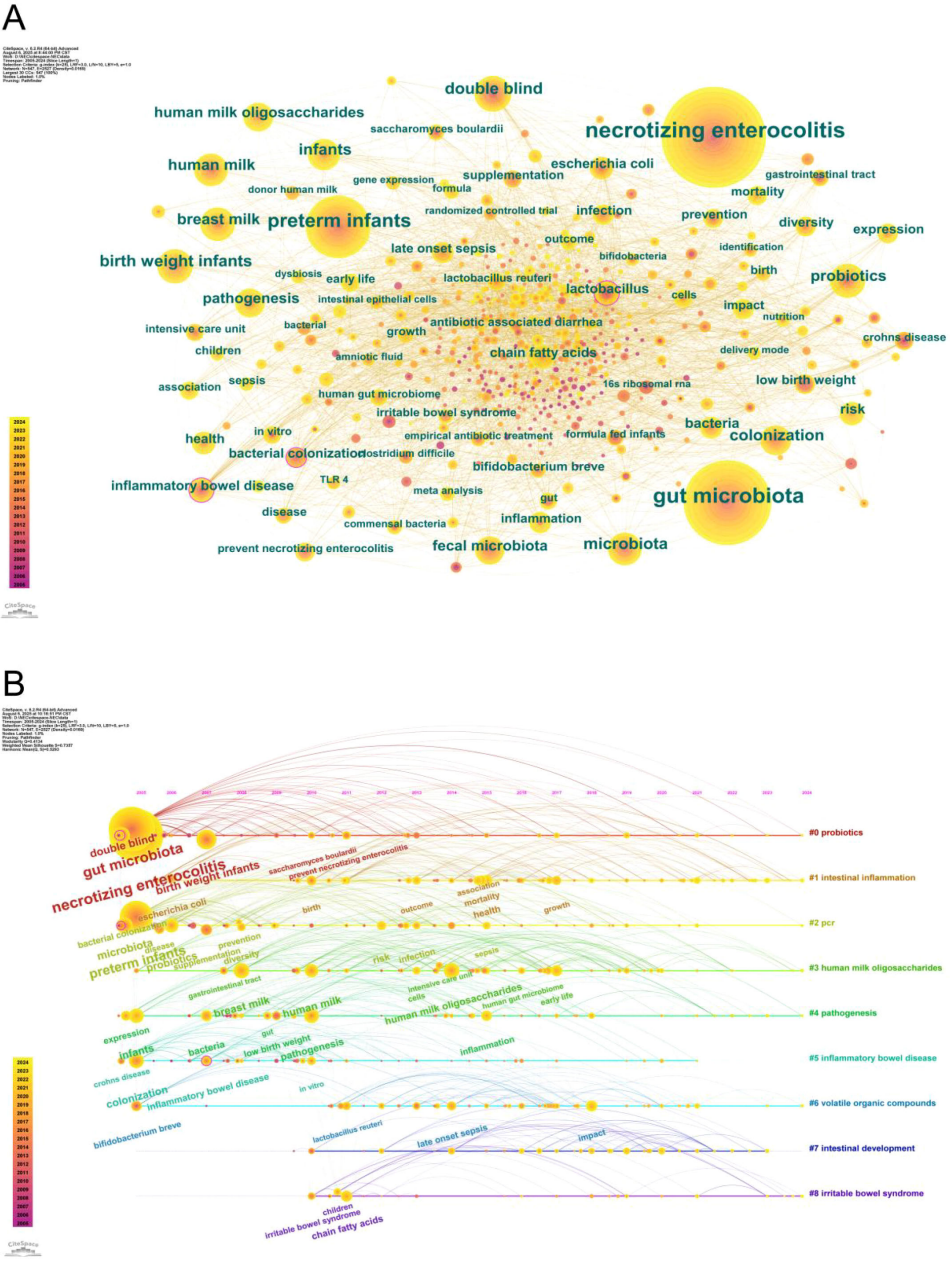
**(A)** Cooperation map of keywords. **(B)** The timeline of clustered network map of author keywords in gut microbiota and NEC research.

We extracted 547 keywords from 1,011 articles. Based on Zipf’s law, keywords appearing more than 16 times were defined as core keywords, and this threshold corresponds to the inflection point at which the cumulative frequency distribution begins to plateau, indicating a transition from core to scattered vocabulary. A total of 72 keywords met this threshold. As illustrated in the keyword co-occurrence network diagram (**Figure 7**), these 72 core keywords were analyzed. The size of each node corresponds to the frequency of the keyword’s occurrence—larger nodes indicate higher frequency.

The timeline diagram displays the most frequently occurring keywords within each cluster over time. **Figure 7**, presenting a timeline analysis of keywords in the NEC and gut microbiota research domain, demonstrates sustained scholarly interest in this field. Cluster #0: probiotics, keywords include “necrotizing enterocolitis,” “infant,” “gut microbiota,” “*Saccharomyces boulardii*,” and “prevention of necrotizing enterocolitis.” The central interest is understanding how probiotics work to prevent NEC by modulating the gut microbiota. Cluster #1: gut inflammation, keywords include “*Escherichia coli*,” “health outcome,” “mortality,” and “health.” This cluster investigates the impact of gut inflammation on health outcomes, with *Escherichia coli* identified as a primary pathogenic driver. Cluster #2: PCR analysis, keywords include “microbiota,” “preterm infant,” “probiotics,” “infection,” and “sepsis.” This cluster focuses on compositional analysis of gut microbiota via PCR and its health implications. Cluster #3: HMOs, keywords include “gastrointestinal tract,” “intensive care unit,” and “cell.” This cluster explores the effects and mechanisms of HMOs on the gastrointestinal tract in critically ill children. Cluster #4: pathogenesis, keywords include “breast milk,” “HMOs,” “low birth weight,” and “gut microbiota.” This cluster examines the mechanisms of HMO-mediated gut microbiota modulation in breastfed low birth weight infants. Cluster #5: inflammatory bowel disease (IBD), keywords include “intestinal inflammation,” “Crohn’s disease,” and “*in vitro*.” This cluster addresses the gut microbiota’s role in inflammatory bowel diseases. Cluster #6: volatile organic compounds (VOCs), keywords include “*Bifidobacterium breve*,” “*Lactobacillus reuteri*,” “late-onset sepsis,” and “impact.” This cluster investigates the effects of gut probiotics on late-onset sepsis. Cluster #7: gut development, keywords not listed. Cluster #8: irritable bowel syndrome (IBS), keywords include “children,” “IBS,” and “chain fatty acids.” This cluster explores the potential mechanistic links between gut microbiota and its metabolites in the pathogenesis and symptom management of IBS in children.

**Figure 8:**
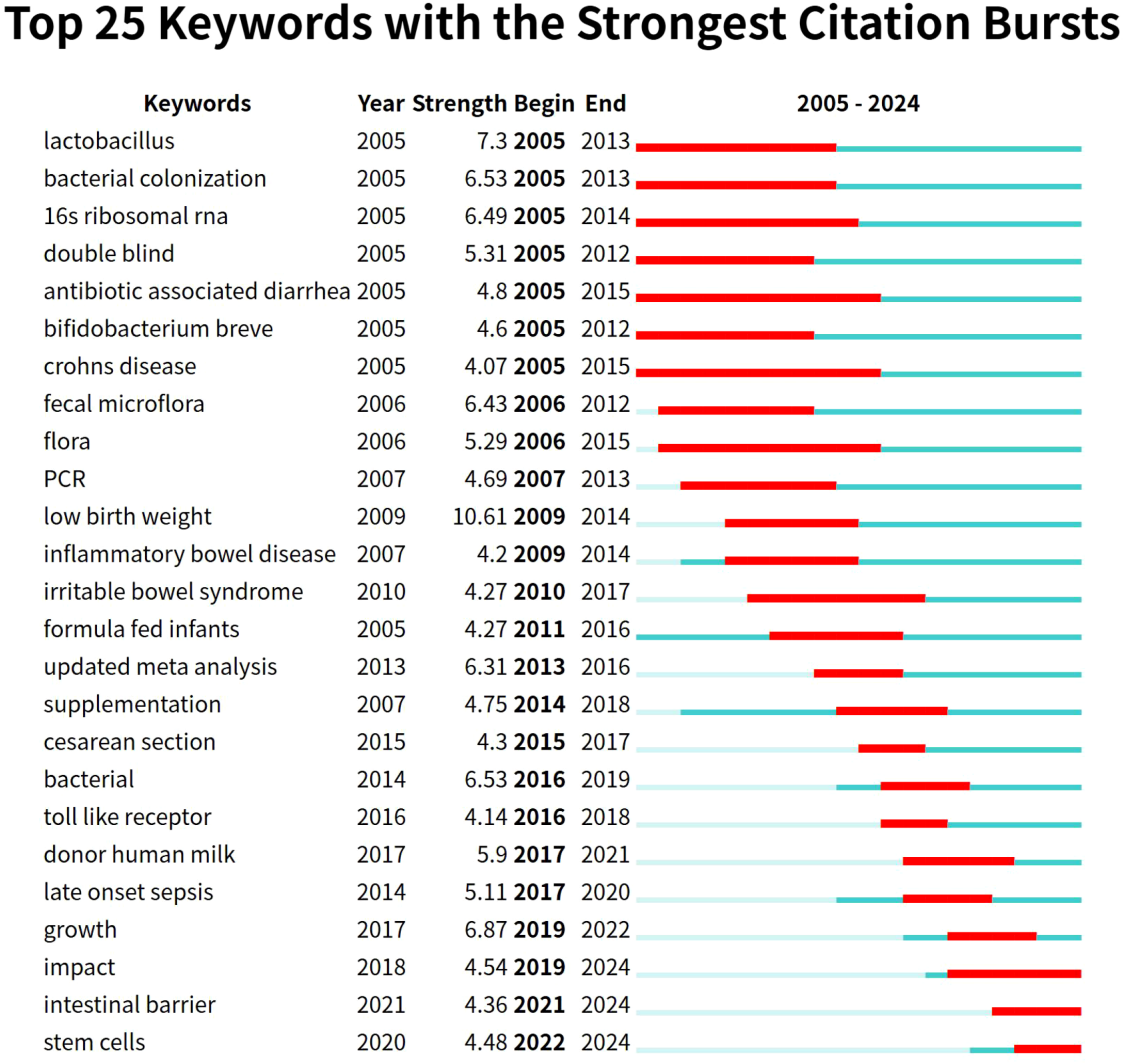
The top 25 keywords with the strongest citation bursts in gut microbiota and NEC research.

Keyword burst graphs (**Figure 8**) can clearly reflect the research hotspots and evolutionary trends within a field. A “burst keyword” refers to a keyword that undergoes a sharp increase in occurrence frequency over a short time period, signaling a sudden surge in research interest.

In **Figure 8**, the red lines indicate the start and end years of a keyword citation burst period, while the green segments represent its duration. As illustrated, during the early research phase (approximately 2005–2013), hotspots were concentrated on analyzing the fundamental composition of the gut microbiota, colonization processes, and the role of specific probiotic strains (*Lactobacillus*, *Bifidobacterium breve*) using methods such as 16S rRNA sequencing and PCR (Wang et al., 2009). As preliminary research accumulated, the field entered an evidence-based phase (approximately 2013–2018), results from numerous double-blind trials were synthesized, and updated meta-analyses were conducted to evaluate the efficacy of interventions like probiotics in preventing NEC (Chang et al., 2017; Pammi et al., 2017). Research further delved into the pathogenesis of NEC and began focusing on the long-term impact of interventions on the growth of preterm infants. Recent research hotspots focus on intestinal barrier repair, stem cell therapy, and the continuous optimization of nutritional interventions (donor human milk, targeted probiotic strategies) (Ganji et al., 2023). The emergence of stem cells as a new keyword with a burst period starting after 2020 indicates a significant future research direction.

### 3.7 Highly cited articles

Citation frequency reflects a paper’s influence and highlights research hotspots within a field. **Table 5** lists the top 10 most cited articles in this domain. The most cited publication is a 2019 review in *Microorganisms* entitled “What is the healthy gut microbiota composition? A changing ecosystem across age, environment, diet, and diseases,” which has been cited 2,181 times to date. Its core argument posits that gut microbiota composition is shaped by both infant factors (delivery mode, feeding type, weaning period) and postnatal factors (diet, antibiotics, lifestyle). The review emphasizes that restoring microbial balance through dietary interventions or microbiome-targeted therapies represents a critical future direction for disease treatment (Meng et al., 2020). Analysis of these 10 articles reveals the field’s current priorities: Gut dysbiosis serves as the pathological basis for NEC and other diseases; future research needs to decipher strain-specific functions of gut microbes; and advancements in personalized microbiome-targeted therapeutics are essential.

**Table 5:** The top 10 cited articles.

Rank	Title	Authors	Source title	Publication year	Total citations	Average per year
1	What is the healthy gut microbiota composition? A changing ecosystem across age, environment, diet, and diseases	Rinninella, E.	Microorganisms	2019	2181	311.57
2	Development of the human infant intestinal microbiota	Palmer, Chana	PLOS Biology	2007	2106	110.84
3	Human milk oligosaccharides: every baby needs a sugar mama	Bode, Lars	Glycobiology	2012	1320	94.29
4	The infant microbiome development: mom matters	Mueller, Noel T.	Trends in Molecular Medicine	2015	671	61
5	The intestinal microbiome in early life: health and disease	Arrieta, Marie-Claire	Frontiers in Immunology	2014	669	55.75
6	Probiotics and the gut microbiota in intestinal health and disease	Gareau, Melanie G.	Nature Reviews Gastroenterology & Hepatology	2010	667	41.69
7	Negative effects of a high-fat diet on intestinal permeability: a review	Rohr, Michael W.	Advances in Nutrition	2020	526	87.67
8	Mechanisms and consequences of intestinal dysbiosis	Weiss, G. Adrienne	Cellular and Molecular Life Sciences	2017	474	52.67
9	Intestinal dysbiosis in preterm infants preceding necrotizing enterocolitis: a systematic review and meta-analysis	Pammi, Mohan	Microbiome	2017	474	52.67
10	Fecal microbiota in premature infants prior to necrotizing enterocolitis	Mai, Volker	PLOS One	2011	471	31.4

## 4 Discussion

The correlation analysis between gut microbiota and NEC has been a research hotspot in recent years. This study represents the first bibliometric analysis in this field. Using software tools such as CiteSpace and VOSviewer, we visually present research hotspots and evolutionary trends in NEC and gut microbiota from 2005 to 2024 across multiple dimensions, providing a visual foundation for rapid comprehension of this domain.

### 4.1 Analysis of countries, institutions, journals, and authors

Research on gut microbiota and NEC has experienced exponential growth over the past two decades, underscoring its significance in the scientific community.

Analysis of national and institutional contributions reveals the following: United States dominance: the country leads in publication volume and influence. The top 3 institutions—University of California System, State University System of Florida, and University of Florida—are all United States-based. The robust collaboration between UC Davis and the University of Chicago accelerates knowledge translation. China ranks second with 158 publications but exhibits lower citation impact (22.65 citations/article), indicating a need to enhance global visibility. China’s output-impact gap: The country ranks second with 158 publications but lower citation impact (22.65 citations/article). European synergy: Countries like the UK and Italy form secondary research clusters through regional collaboration, enhancing knowledge integration.

Journal distribution highlights interdisciplinary convergence: “Microbiome” (IF = 12.7) leads in basic research. “Nutrients” and “Frontiers in Immunology”reflect multidisciplinary engagement. “Microorganisms” achieves the highest citation impact (130.84 citations/article).

Author collaboration networks center on Mark A. Underwood (31 publications, h-index 23) and Josef Neu (high average citations: 107.88/article). Their collaboration, along with institutions like the University of California and University of Copenhagen, drives pivotal research on interventional mechanisms of breast milk components.

### 4.2 Research hotspots and trends

Through keyword clustering, timeline analysis, and examination of highly cited literature, we identify the evolving research hotspots and trends in this field. Studies on gut microbiota and NEC have shifted from initially characterizing microbial composition toward mechanism-driven multi-omics integrated analysis, progressively advancing toward clinical translation.

In the early research phase, 16S rRNA gene sequencing analyses of preterm infant fecal samples revealed a significant reduction in gut microbiota diversity prior to NEC onset. Concurrently, Proteobacteria (particularly Enterobacteriaceae such as *E. coli* and *Klebsiella*) exhibited overproliferation, while Firmicutes and Bacteroidetes decreased. These findings established microbial dysbiosis (characterized by low diversity + Proteobacteria dominance) as a core feature of NEC (Patel and Denning, 2015; Cassir et al., 2016).

Mid-phase research focused on elucidating mechanisms by which gut microbiota influence NEC pathogenesis, with integrative analyses of TLR4 signaling, intestinal barrier integrity, and breast milk components revealing that overgrowth of Proteobacteria activates the TLR4/NF-κB pathway, triggering pro-inflammatory cytokines (TNF-α, IL-6) that disrupt gut barrier integrity (Neu and Pammi, 2018); concurrently, microbiota-derived metabolites—including short-chain fatty acids (SCFAs), tryptophan derivatives, and bile acids—play pivotal roles in NEC pathogenesis by modulating barrier function, immune responses, and microbial composition (Liu et al., 2022; Kaplina et al., 2023b), where, specifically, reduced butyrate levels (due to altered microbiota and diminished fiber fermentation) compromise barrier integrity, increase permeability, and promote bacterial translocation. This conceptual shift from taxonomic identification to functional interrogation of microbiota marks a paradigm shift toward mechanistic depth in NEC research.

Metagenomics provides enhanced insights into the neonatal gut microbiome, informing the potential of probiotics, prebiotics, and synbiotics as preventive and therapeutic measures. Probiotics/prebiotics (particularly *Bifidobacterium* and *Lactobacillus* complexes) and bioactive breast milk components (HMOs, lactoferrin) mitigate dysbiosis and prevent NEC by regulating microbiome development. Meta-analyses further indicate that probiotic formulations containing *Lactobacillus* alone or combined with *Bifidobacterium* reduce the incidence of severe NEC (Vandenplas et al., 2018; Chi et al., 2021), while HMOs confer protection by promoting *Bifidobacterium* colonization and stimulating short-chain fatty acid production (Nolan et al., 2020; Sun et al., 2024). Animal studies demonstrate that fecal microbiota transplantation restores microbial balance and alleviates inflammation, offering novel approaches for individualized NEC prevention (Brunse et al., 2022).

It is important to note that not all intervention studies have reported positive outcomes. While meta-analyses have demonstrated that multistrain probiotics reduce the incidence of severe NEC and all-cause mortality in preterm infants (Chang et al., 2017; Chi et al., 2021), some large randomized controlled trials found no significant benefit with specific single-strain preparations, and rare cases of probiotic-associated sepsis have been documented in immunocompromised neonates (Underwood et al., 2020). Similarly, although HMOs and lactoferrin show protective effects in observational studies, randomized trials of bovine lactoferrin supplementation have yielded mixed results, with some showing no reduction in NEC risk (Vandenplas et al., 2018). These contradictory findings highlight the need for strain-specific, context-aware interventions. Our bibliometric analysis captured these debates through keyword bursts such as “meta-analysis” (2013–2018) and “randomized controlled trial,” reflecting the field’s ongoing effort to reconcile positive and negative evidence. Acknowledging both successful and unsuccessful interventions is crucial for avoiding overoptimistic conclusions and for guiding future research toward resolving these discrepancies.

### 4.3 Limitations

Due to the stringent requirements and standards of bibliometric analysis software for data quality, this study exclusively selected English-language articles and review articles from the WoSCC to ensure data integrity. Consequently, publications not indexed in this database and non-English literature may have been omitted. Despite these limitations, the analytical results derived from the included data demonstrate broad consistency with existing research on NEC and gut microbiota.

## 5 Conclusions

This study employs bibliometric analysis to systematically delineate the knowledge landscape of gut microbiota and NEC research from 2005 to 2024. The data reveal a consistent growth trajectory in this field, reflecting its escalating academic significance. The United States leads in research output, demonstrated by high productivity, citation impact, and extensive collaboration networks. Key advancements evolved through three distinct phases: microbiome structural characterization (primarily driven by 16S rRNA technology), multi-omics exploration of underlying mechanisms (focusing on TLR4 signaling/metabolite pathways), and transition toward clinical translational research (probiotic/breast milk component interventions).

## Data Availability

The original contributions presented in the study are included in the article/supplementary material. Further inquiries can be directed to the corresponding author.
